# Magnetic resonance analysis of deep cerebral venous vasospasm after subarachnoid hemorrhage in rabbits

**DOI:** 10.3389/fcvm.2022.1013610

**Published:** 2022-09-21

**Authors:** Zixuan Zhang, Qiong Fang, Yu Zhang, Youzhi Zhu, Wei Zhang, Youyou Zhu, Xuefei Deng

**Affiliations:** ^1^Department of Clinical Medicine, West Anhui Health Vocational College, Lu'an, China; ^2^Department of Anatomy, Anhui Medical University, Hefei, China; ^3^Department of Basic Medicine, Anhui Medical College, Hefei, China; ^4^Department of Radiology, The 901st Hospital of the Joint Logistics Support Force of PLA, Hefei, China

**Keywords:** subarachnoid hemorrhage, cerebral vasospasm, deep cerebral vein, basilar artery, magnetic resonance imaging

## Abstract

**Objective:**

Arterial spasm is proved to be an inducer of cerebral ischemia and cerebral infarction, while when a venous spasm occurs, cerebral edema is seen to be caused by a disturbance in cerebral blood flow. However, it is unclear and unproven whether venous spasm occurs after subarachnoid hemorrhage (SAH). To provide the theoretical basis for treating cerebral vasospasm after SAH, magnetic resonance imaging (MRI) was employed to observe the changes in the diameter of deep cerebral veins in rabbits after SAH.

**Methods:**

Fourteen New Zealand rabbits were randomly divided into the SAH group (*n* = 10) and the normal saline group (NS group, *n* = 4). Specifically, the SAH models were established by the ultrasound-guided double injections of blood into cisterna magna. Moreover, the MRI was performed to observe the changes in the diameter of deep cerebral veins (internal cerebral vein, basilar vein, and great cerebral vein) and basilar artery before modeling (0 d) and 1, 3, 5, 7, 9, and 11 d after modeling.

**Results:**

In the SAH group, the diameter of the basilar artery showed no evident change on the 1st d. However, it became narrower obviously on the 3rd d and 5th d, and the stenosis degree was more than 30%. The diameter gradually relieved from 7th to 9th d, and finally returned to normal on the 11th d. Moreover, the diameter of the internal cerebral vein significantly narrowed on the 1st d, the stenosis degree of which was 19%; the stenosis then relieved slightly on the 3rd d (13%), reached the peak (34%) on the 5th d, and gradually relieved from 7th d to 11th d. Moreover, the stenosis degree of the basilar vein was 18% on the 1st d, 24% on the 3rd d, and reached the peak (34%) on the 5th d.

**Conclusion:**

After SAH in rabbits, the cerebral vasospasm was seen to occur in the basilar artery, and likewise, spasmodic changes took place in the deep cerebral vein. Furthermore, the time regularity of spasmodic changes between the cerebral vein and basilar artery was of significant difference, indicating that the venous vasospasm resulted in active contraction.

## Introduction

Subarachnoid hemorrhage (SAH) refers to the blood flowing into the subarachnoid space after the rupture of intracranial vessels, which is a common clinical critical disease (1). Although surgical technology and scientific research develop at a fast rate, the mortality and disability rate of SAH have not yet been effectively controlled (2). For instance, delayed cerebral vasospasm is a common and serious complication after SAH, which is manifested as the continuous contraction of the smooth muscle of one or more intracranial vessels, or the morphological changes of lumen caused by vascular injury, and the stenosis of the vascular lumen during angiography (3). Moreover, persistent stenosis is seen to be an inducer of increased intracranial pressure, neurological function defect, delayed cerebral ischemia, and poor clinical outcome (4). The patient's symptoms, however, worsen after treatment, thus prolonging the length of hospital stay, increasing medical costs, and becoming the leading cause of disability and death (5).

The pathogenesis of cerebral vasospasm has been widely researched in previous studies (6–10). It is generally believed that cerebral vasospasm after SAH is caused by multiple factors acting on the intracranial arteries, including various physical and chemical stimuli. However, it is not clear whether the intracranial venous spasm would also occur after SAH. Compared with the cerebral arterial system, the cerebral venous system is usually asymmetric, highly variable, and complex in three-dimensional conformation. These characteristics resulted in the fact that more focus has been placed for years on the cerebral artery instead of the cerebral vein (11). However, due to the rapid improvement of medical diagnosis and treatment and continuous development of imaging technology, the role of the morphological characteristics of cerebral veins has gained increasing emphasis in the regulation of cerebral blood circulation and the occurrence and development of cerebral venous diseases (12–15).

Vascular smooth muscle cells are the ultimate target cells for vasoconstriction spasms. Traditionally, the wall of the cerebral vein is believed to lack smooth muscle cells, which play only a passive drainage role in cerebral blood circulation (16). However, it has been confirmed by histological studies that smooth muscle cells also exist in the wall of the cerebral vein (17). It follows that cerebral veins have the histomorphological basis of vasospasm. From another point of view, both the cerebral artery and cerebral vein are located in subarachnoid space. In this way, when this space is full of blood, and when blood and active vascular substances act on the cerebral artery, it might be able to affect the cerebral vein further. In other words, factors leading to cerebral arterial spasm also act on the cerebral vein, possibly leading to contraction and spasm.

If vasospasm occurs both in the cerebral vein and cerebral artery after SAH, it might be an inducer of the brain edema. Moreover, due to the different occurrence times, the cerebral edema caused by cerebral venous obstruction should be different in treatment from that caused by cerebral artery contraction. Specifically, patients with cerebral artery systolic spasms mainly adopt the treatment of increasing cerebral perfusion pressure and vasodilation (18). Due to the poor venous return, the treatment measures of increasing cerebral perfusion pressure will lead to further aggravation of cerebral edema. Accordingly, cerebral vasoconstriction spasms can only be treated by vasodilation therapy. Therefore, with respect to the treatment options for the patients, it is of great significance to accurately grasp the time of spasm occurrence, and to figure out whether the cerebral artery or the cerebral vein is contracted after SAH.

As it is difficult to carry out *in vivo* experimental studies on cerebral vasospasm and pathological changes in brain tissue after SAH, and as the materials provided by clinical autopsy are extremely limited, animal models have become one of the primary means of studying cerebral vasospasm (19). In the present study, ultrasound-guided double injections of blood into cisterna magna were used to make the SAH model in rabbits. The operation was simple, the experimental animal achieved zero death, and the vasospasm effect was evident after modeling (20). The collateral circulation of a deep cerebral vein is less, and its vasospasm will lead to an extensive range of cerebral parenchymal blood reflux obstruction. Moreover, as shown in the research, the incidence of brain parenchyma injury in patients with deep cerebral venous disease was significantly increased, the consequences were more serious, and the mortality rate was as high as 15% (21). Therefore, this study attempts to apply magnetic resonance imaging (MRI) technology to observe the changes in the diameter of the cerebral artery and cerebral vein after SAH in rabbits to provide a theoretical basis for treating cerebral vasospasm.

## Materials and methods

### Experimental grouping and model establishment

The study was approved by the Ethics Committee of Anhui Medical University (Hefei, China, 2012238). Moreover, the animal use and care protocols, including the operation procedures, were conducted strictly according to the *Guide for the Care and Use of Laboratory Animals of the National Institutes of Health*.

A total of 14 adult male New Zealand rabbits, weighing from 2.5 kg to 3.2 kg, were supplied by the Experimental Animal Center of Anhui Medical University. They were then randomly divided into the SAH group (*n* = 10) and the normal saline (NS) group (*n* = 4) separately. Specifically, in the SAH group, the SAH model was established by ultrasound-guided double injections of blood into cisterna magna, and the procedure followed has been described in the previous report (20).

### Magnetic resonance imaging (MRI) scanning

The MRI was performed before (0 d) and 1, 3, 5, 7, 9, and 11 d after modeling. After being anesthetized, New Zealand rabbits were placed in the left decubitus position and examined with 3.0T MRI equipment (SIEMENS TRIO) with a knee coil (similar in size to rabbit). Moreover, cerebral venous images were scanned with susceptibility-weighted imaging (SWI). Specifically, the technical parameters were as follows: FOV 120 mm, slice thickness 0.6 mm, TR 29 MS, TE 20 ms, and average 8. Furthermore, the arterial scanning sequence was time-of-flight magnetic resonance angiography (TOF-MRA), while the technical parameters were listed as follows: FOV 110 mm, slice thickness 0.5 mm, TR 25 ms, TE 5.72 ms, and average 5. After scanning, the data were then sent to the workstation for minimum intensity projection (MIP) and 3D-TOF-MRA.

### Evaluation of vasospasm

The MRI images were analyzed by OsiriX 3.6 (MAC OS 10.5.8) software and measured by two experienced radiologists *via* a double-blind method. Following this, the diameter of the internal cerebral vein and the basilar vein was measured, in the first place, by the mean value of the bilateral origin part. Next, the diameter of the great cerebral vein and basilar artery was measured in the middle part of the vessel. The stenosis degree and the change rate of different vessels were then calculated in accordance with the standard of Liszczak (22). The calculation formula was as follows:


(1) $$\begin{array}{l} \text{Stenosisdegree}=\left({\text{D}}_{0}-{\text{D}}_{\text{n}}\right)\times 100\text{\%}/{\text{D}}_{0} \end{array}$$

In the formula, D_0_ represents the diameter before modeling, while D_n_ refers to the diameter on n d (*n* = 1, 3, 5, 7, 9, 11) after modeling in the same rabbit.


(2) $$\begin{array}{l} \text{Changerate}=\left({\text{D}}_{\text{n}}-{\text{D}}_{\text{n}-1}\right)\times 100\text{\%}/{\text{D}}_{\text{n}-1} \end{array}$$

In the formula, D_n_ refers to the diameter on n d (*n* = 1, 3, 5, 7, 9, 11) after modeling in the same rabbit; D_n − 1_, however, represents the diameter in the previous time.

### Statistical analysis

All the diameters were inputted in the Excel spreadsheet, and the SPSS 23 statistical software package was used to calculate the difference. Additionally, the diameter was expressed as mean ± standard deviation ($\bar{x}$ ± s), while the comparison of multiple groups was conducted by single-factor analysis of variance.

## Results

### MR imaging of cerebral vessels

**Figure 1:**
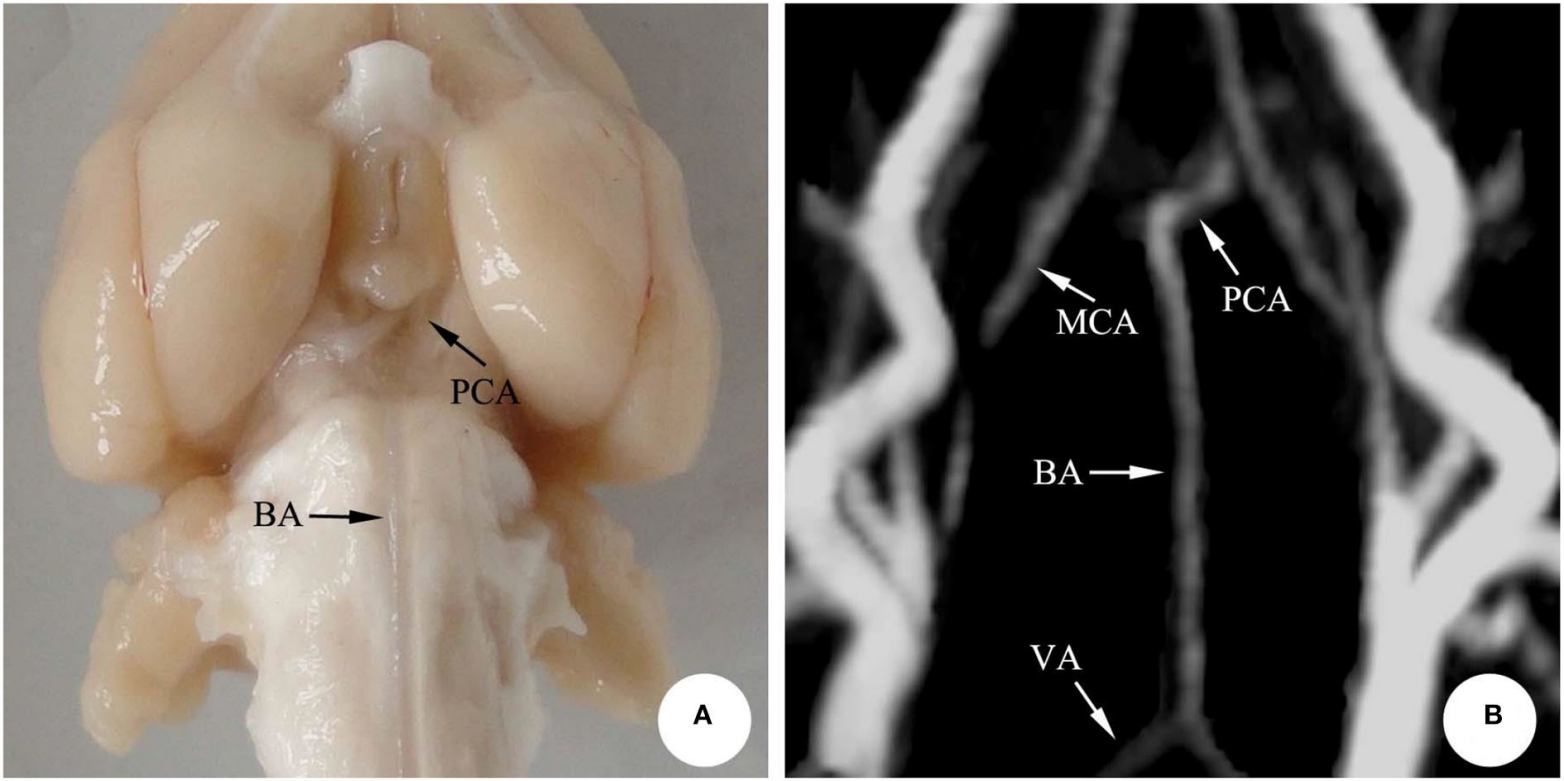
The basilar artery in rabbit brain. **(A)** Microscopic anatomy; **(B)** 3D-TOF-MRA. BA, Basilar artery; PCA, Posterior cerebral artery; VA, Vertebral artery; MCA, Middle cerebral artery.

According to the microscopic anatomy of the rabbit brain, the basilar artery, running along the basal sulcus of the ventral pontine, fell into the left and the right posterior cerebral arteries from the superior border of the pons. Moreover, in accordance with the 3D-TOF-MRA images, the arteries showed striped hyperintensity, with the left and the right vertebral arteries joining the basilar artery at the junction of pons and medulla oblongata. The basilar artery, however, ascended and fell into the left and the right posterior cerebral arteries, finally forming the middle and posterior parts of the cerebral artery ring together with the middle cerebral artery. The morphology of the basilar artery was consistent with that of MRI (Figure 1).

**Figure 2:**
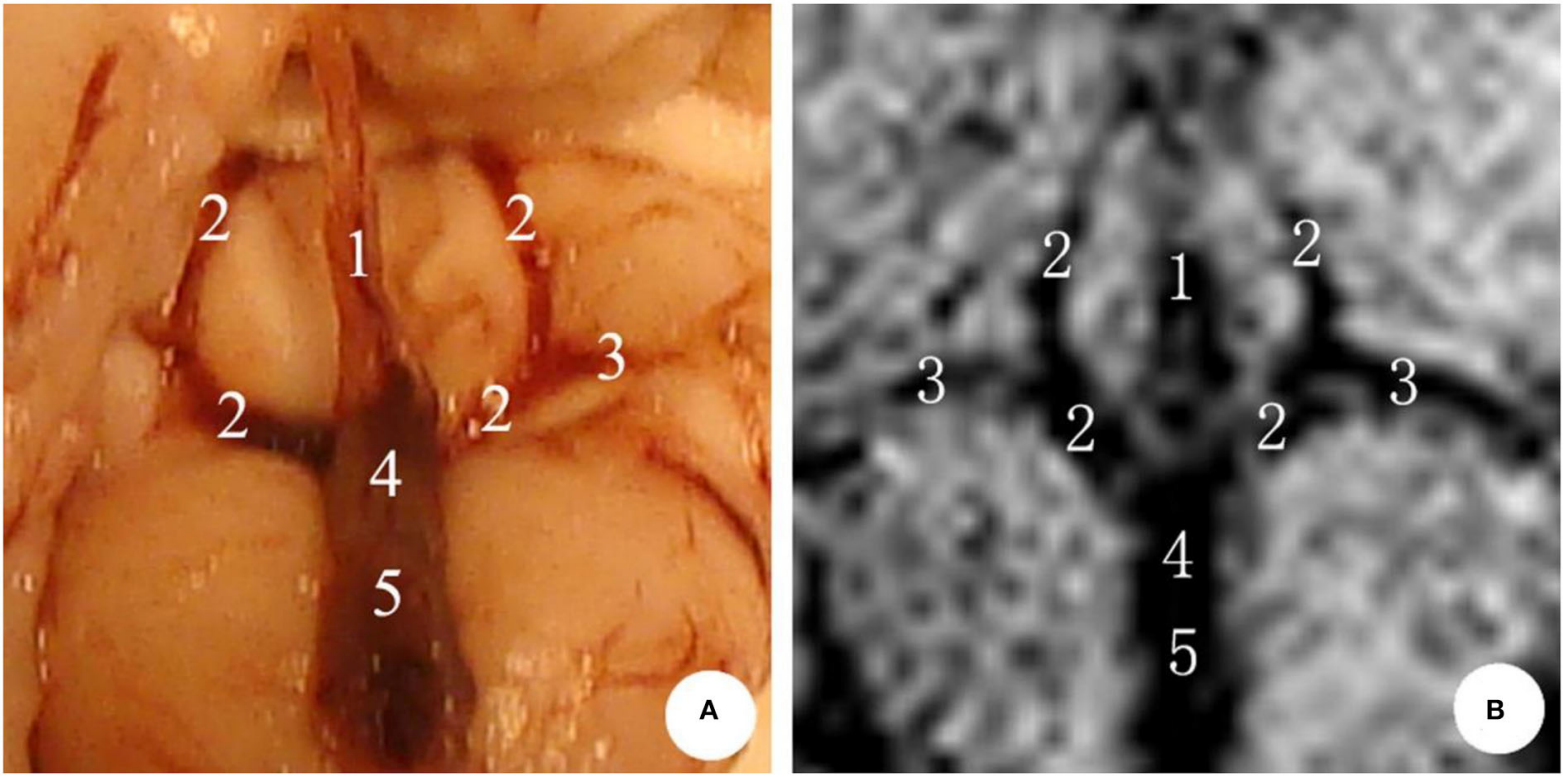
The deep cerebral veins in rabbit brain. **(A)** Microscopic anatomy; **(B)** MIP image in MRI. 1: inferior sagittal sinus; 2: internal cerebral vein; 3: basal vein; 4: great cerebral vein; 5: straight sinus.

In the microscopic anatomy of the rabbit brain, the bilateral internal cerebral veins went from front to back, received the venous blood from both basilar veins, and then flowed into the great cerebral vein. Finally, the great cerebral vein and the inferior sagittal sinus entered into the straight sinus, where no obvious boundary was seen between the great cerebral vein and the straight sinus. Following this, the original image was reconstructed by MIP *via* SWI sequence scanning, and the deep cerebral veins of rabbits were visible, showing hypointensity. It follows that compared with microanatomy, the shape of the deep cerebral veins was consistent (Figure 2).

### Diameter of cerebral vein and artery

#### Basilar artery

The mean diameter of the basilar artery in the NS group was (0.66 ± 0.04) mm on 0 d, and there was no significant difference during 1st−11th d after modeling and before modeling (*P* > 0.05).

**Figure 3:**
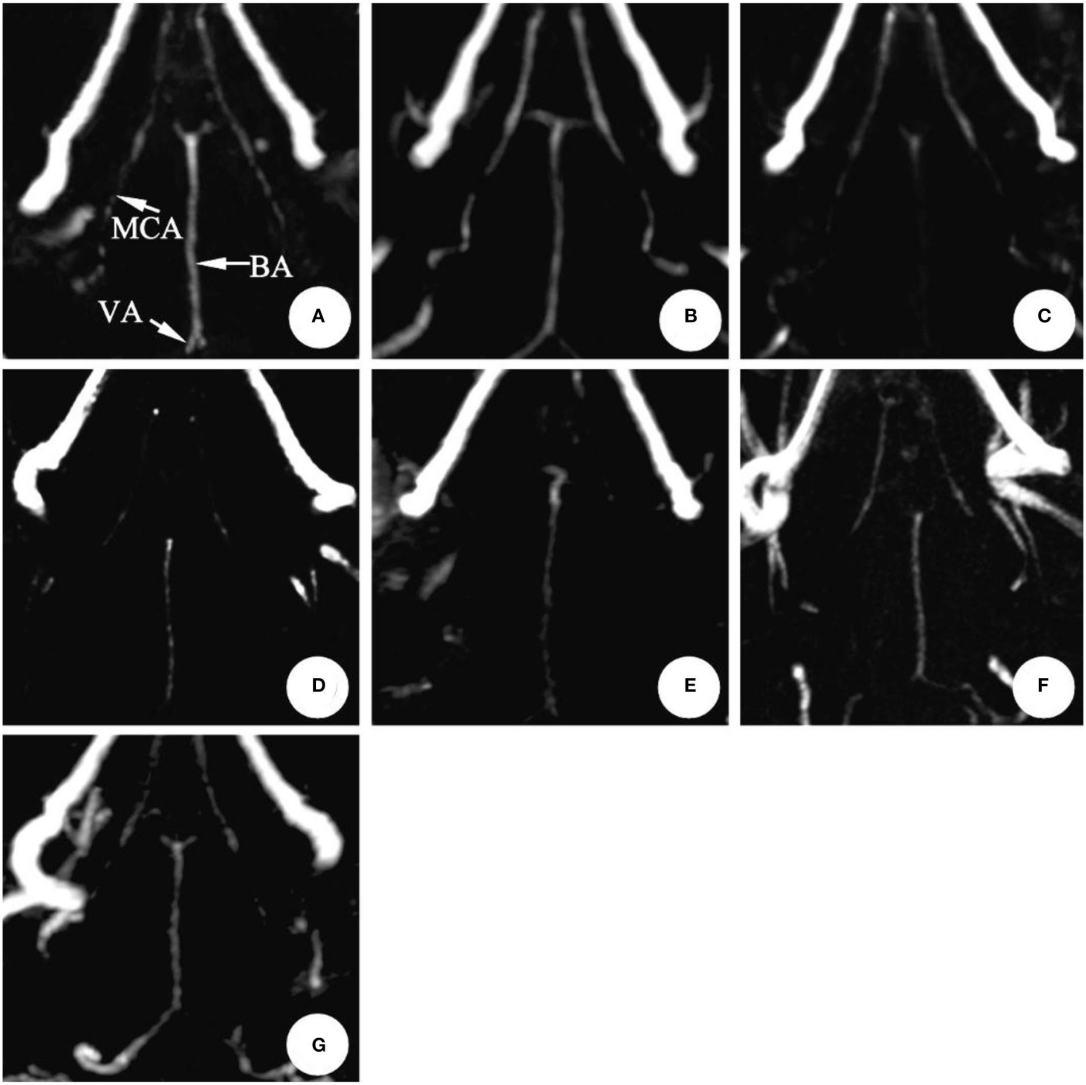
The magnetic resonance performance of artery in the same rabbit SAH model within 0 ~ 11 d (3D-TOF-MRA). The diameter showed no significant changes after making the model in 1 d. It reached the peak in 3~5 d and gradually recovered during 7~11 d.BA, Basilar artery; MCA, Middle cerebral artery; VA, Vertebral artery. **(A)** 0 d; **(B)** 1 d; **(C)** 3 d; **(D)** 5 d; **(E)** 7 d; **(F)** 9 d; **(G)** 11 d.

In the SAH group, the mean diameter of the basilar artery was (0.66 ± 0.06) mm on 0 d and was (0.64 ± 0.06) mm on 1st d, and there was no significant difference between the two groups (*P* > 0.05); the basilar artery showed obvious spasm on 3rd d (6 cases) and 5th d (4 cases) after modeling, and the average diameter was (0.42 ± 0.10) mm and (0.44 ± 0.07) mm, respectively (*P* < 0.05); after 7th d, it began to relieve and reached normal on 11th d (*P* > 0.05) (Table 1, Figure 3).

**Table 1:** The diameter of the basilar artery and deep cerebral veins.

**Item**	**0 d**	**1 d**	**3 d**	**5 d**	**7 d**	**9 d**	**11 d**	***F*/*P***
Basilar artery								
SAH group	0.66 ± 0.06	0.64 ± 0.06	0.42 ± 0.10*	0.44 ± 0.07*	0.57 ± 0.06*	0.60 ± 0.06*	0.66 ± 0.05	22.09/0.00
NS group	0.66 ± 0.04	0.66 ± 0.03	0.67 ± 0.02	0.66 ± 0.02	0.67 ± 0.02	0.65 ± 0.04	0.66 ± 0.03	0.23/0.96
Internal cerebral vein								
SAH group	0.53 ± 0.05	0.43 ± 0.08*	0.46 ± 0.08*	0.35 ± 0.10*	0.38 ± 0.08*	0.45 ± 0.05*	0.51 ± 0.05	8.09/0.00
NS group	0.52 ± 0.02	0.51 ± 0.04	0.50 ± 0.01	0.50 ± 0.02	0.51 ± 0.02	0.50 ± 0.01	0.50 ± 0.02	0.66/0.68
Basal vein								
SAH group	0.38 ± 0.08	0.31 ± 0.07*	0.29 ± 0.09*	0.25 ± 0.12*	0.27 ± 0.08*	0.30 ± 0.06*	0.36 ± 0.06	10.21/0.00
NS group	0.39 ± 0.05	0.39 ± 0.03	0.38 ± 0.04	0.39 ± 0.05	0.38 ± 0.04	0.39 ± 0.03	0.38 ± 0.03	0.51/0.88
Great cerebral vein								
SAH group	1.33 ± 0.31	1.32 ± 0.28	1.29 ± 0.29	1.26 ± 0.31	1.30 ± 0.32	1.31 ± 0.33	1.32 ± 0.34	0.63/0.72
NS group	1.32 ± 0.29	1.32 ± 0.29	1.32 ± 0.29	1.32 ± 0.29	1.32 ± 0.29	1.32 ± 0.29	1.32 ± 0.29	0.45/0.83

* P < 0.05 vs. 0 d.

#### Internal cerebral vein

The mean diameter of the internal cerebral vein in the NS group was (0.52 ± 0.02) mm on 0 d, and there was no significant difference between 1 and 11 d after modeling and before modeling (*P* > 0.05).

**Figure 4:**
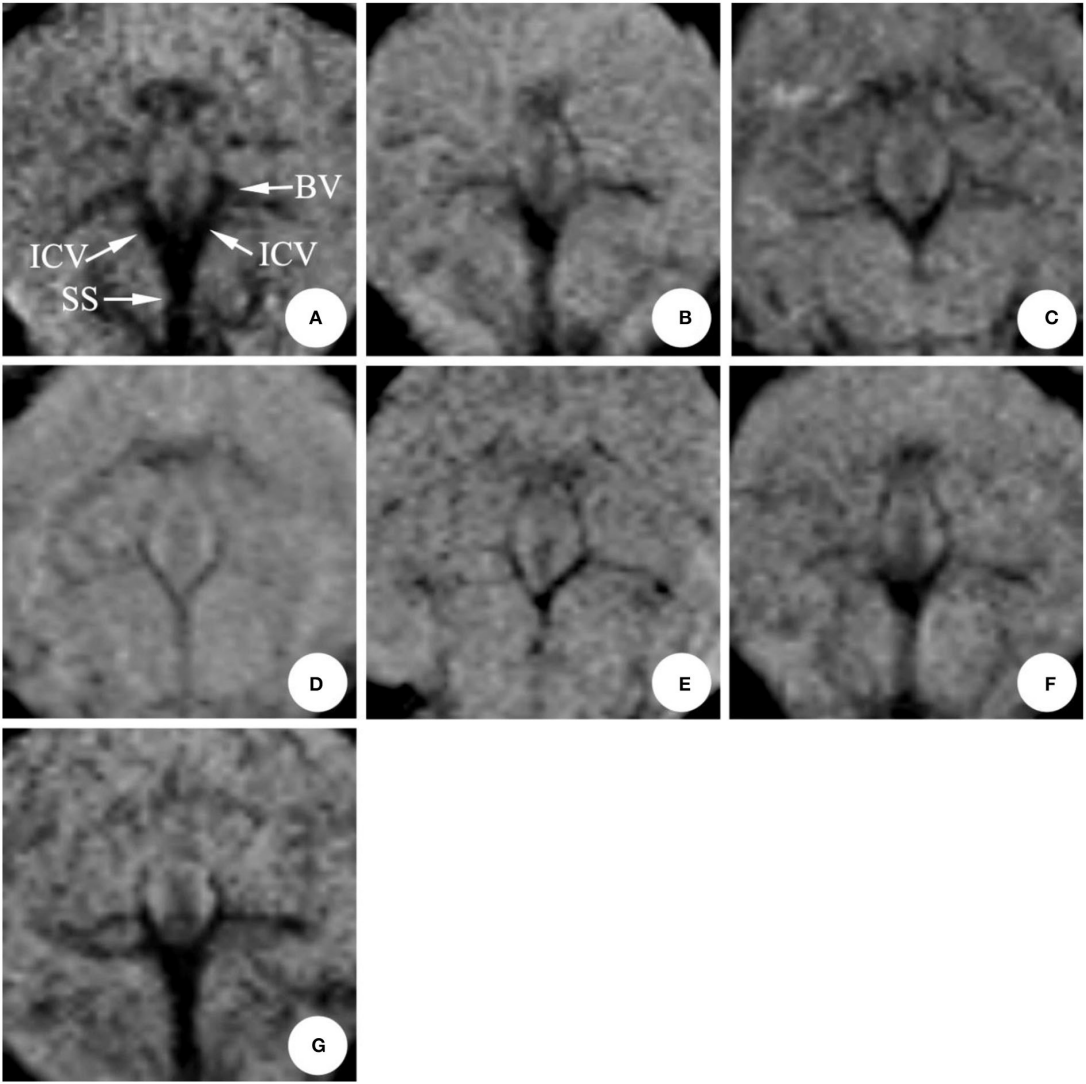
The magnetic resonance performance of internal cerebral vein in the same rabbit SAH model within 0 ~ 11 d (mIP). The diameter was obviously narrow after making the model in 1 d, eased after 3 d, and reached a peak around 5~7 d, and gradually recovered during 9~11 d. ICV, Internal cerebral vein; SS, Straight sinus; BV, Basilar vein. **(A)** 0 d; **(B)** 1 d; **(C)** 3 d; **(D)** 5 d; **(E)** 7 d; **(F)** 9 d; **(G)** 11 d.

In the SAH group, the mean diameter of the internal cerebral vein was (0.53 ± 0.05) mm on 0 d; the mean diameter was (0.43 ± 0.08) mm on 1st d after modeling, and the difference was statistically significant (*P* < 0.05); the stenosis was relieved on 3rd dafter modeling; the stenosis degree reached the peak on 5th d (six cases) and 7th d (four cases), respectively, with the average diameter of (0.35 ± 0.10) mm. There was no significant difference between the two groups (*P* > 0.05) (Table 1, Figure 4).

#### Basilar vein

In the NS group, the mean diameter of the basilar vein was (0.39 ± 0.05) mm on 0 d, and there was no significant difference between 1 and 11 d after modeling and before modeling (*P* > 0.05).

In the SAH group, the mean diameter of the basilar vein was (0.38 ± 0.08) mm before modeling, and the stenosis was (0.31 ± 0.07) mm on 1st d after modeling, and the difference was statistically significant (*P* < 0.05); there was no remission on 3rd d, and the average diameter of the blood vessel was (0.29 ± 0.09) mm; after that, the change rule was consistent with that of the internal cerebral vein. The stenosis degree reached the peak on the 5th d and 7th d, and the mean diameter of vessels was (0.25 ± 0.12) mm and (0.27 ± 0.08) mm, respectively (*P* < 0.05). From 9th d to 11th d, the diameter gradually returned to normal with (0.51 ± 0.05) mm on 11th d (*P* > 0.05) (Table 1, Figure 4).

#### Great cerebral vein

Compared with the data before modeling, the diameter of the great cerebral vein decreased slightly after modeling, but the difference was not statistically significant (Table 1, Figure 4).

### Comparison of stenosis degree

The stenosis degree of the internal cerebral vein was 18.86% on 1st d after modeling, 13.21% on 3rd d, 33.96% on 5th d, and 3.77% on 11th d, respectively.

The stenosis degree of the basilar vein was similar to that of the internal cerebral vein, which was 18.42% on 1st d and 23.68% on 3rd d, respectively. Similarly, the stenosis peaked on 5th d, which was 34.21% compared with the basal value. The stenosis gradually relieved from 7th d to 11th d, which was 5.26% on 11th d.

**Figure 5:**
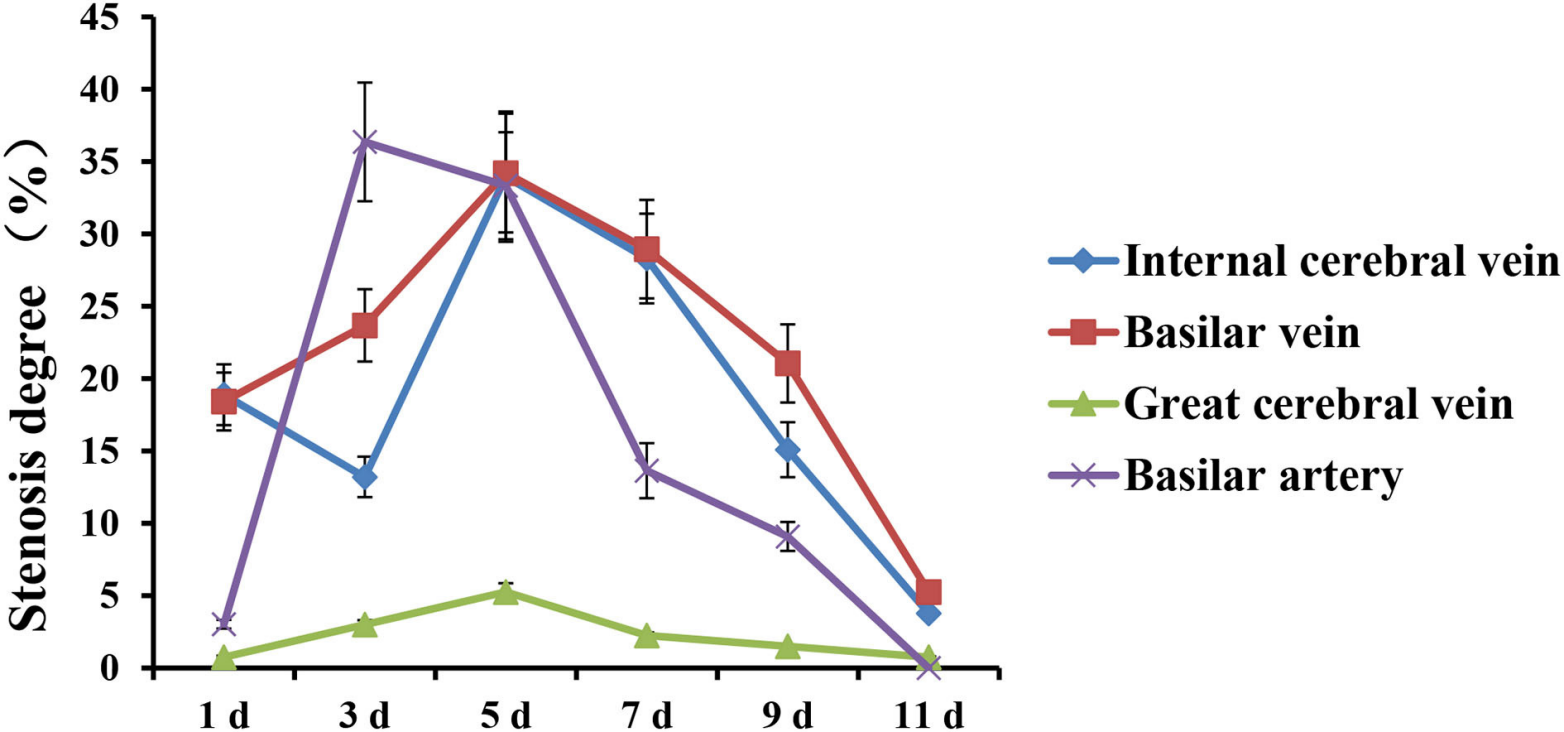
The trends of stenosis degree of deep cerebral veins and basilar artery in rabbit SAH model within 0 ~ 11 d.

Compared with 0 d, the changes of the basilar artery were not obvious on 1st d after modeling, and the degree of stenosis was only 3.03%. The severe and obvious thinning occurred on 3rd d, with the stenosis degree reaching 36.36%. The stenosis degree was still as high as 33.33% on 5th d and gradually relieved from 7th d to 11th d, which were 13.64%, 9.09%, and 0%, respectively (Figure 5).

### Change rate

The diameter of vessels in the SAH group was compared with that of two adjacent measurements. Specifically, the change rate of vessel diameter was obtained by subtracting the measurement value of the last time from the previous measurement value, and then dividing the previous value. Accordingly, if the result is positive, it will be reduced; if it is negative, it will be enlarged.

The positive change rate of the internal cerebral vein and the basilar vein was higher on the 1st d and 5th d. The positive change rate of the basilar artery was higher on the 3rd d (Table 2).

**Table 2:** The change rate of the internal cerebral vein, basilar vein, and basilar artery (%).

**Item**	**1 d**	**3 d**	**5 d**	**7 d**	**9 d**	**11 d**
Internal cerebral vein	18.89	−6.98	23.91	−8.57	−18.42	−13.33
Basal vein	18.42	6.45	13.79	−8.00	−11.11	−20.00
Basal artery	3.03	34.38	−4.76	−29.55	−5.26	−10.00

## Discussion

### Difficulties in research and treatment of cerebral vasospasm

Delayed cerebral vasospasm after SAH was high in the incidence rate and severe in complications, thus prolonging the hospitalization time and increasing the medical expenses. Moreover, it is also considered as one of the critical factors affecting the prognosis of patients. Cerebral vasospasm has always been a hot issue in the field of neurosurgery. Although it has explained many pathophysiological mechanisms of cerebrovascular diseases and provided much evidence and methods for clinical diagnosis and treatment of cerebral vasospasm, the pathogenesis of cerebral vasospasm has not been fully elucidated. Since Ecker et al. (23) first confirmed the phenomenon of cerebral vasospasm after SAH by cerebral angiography, which has attracted extensive attention in neurosurgery. Even though surgical technology and scientific research have improved significantly in recent years, the high mortality and delayed neurological dysfunction of cerebral vasospasm after SAH has not yet been effectively controlled.

For years, clinical practice, animal experiments, basic research, and other scientific methods focused on cerebral arterial spasms, on which the clinical treatment effect is not yet satisfactory. In the clinical data of Vergouwen et al. (24), compared with patients without cerebral arterial spasm, the incidence of cerebral infarction and prognosis of patients with cerebral arterial spasm had no statistical significance. What is more, the study of Al-Khindi et al. (25) also showed that the long-term prognosis of patients with SAH was not ideal, since it may lead to cognitive and neurological impairment. Therefore, such inducers of poor prognoses, such as cerebral vasospasm, needed further study. Accordingly, we speculated that there might exist other factors affecting cerebral vasospasm.

It is generally believed that the collateral circulation of the cerebral vein is abundant. Even if a single or small range of cerebral veins have a spasm, the blood in the drainage area can be relieved through the collateral circulation, which is not likely to cause serious complications. This may be one of the reasons why clinical treatment and researches pay less attention to the cerebral vein. However, the deep cerebral vein is sparse in collateral circulation but large in volume and scale. Suppose that vasospasm is prone to causing a large range of cerebral parenchymal blood flow obstruction and brain edema if vasospasm occurs. Besides, clinical studies also show that, compared with the disease of the superficial cerebral vein system, the incidence of brain parenchyma injury in patients with the deep cerebral vein disease is significantly increased, and the consequences are more serious (21).

In the clinical treatment of cerebral vasospasm, it is necessary to prevent arterial spasms while maintaining cerebral blood perfusion to prevent cerebral edema. The main methods are expanding blood vessels and maintaining perfusion pressure (26). However, cerebral edema caused by cerebral venous reflux varies from that caused by cerebral artery contraction spasm in treatment. More specifically, due to the poor venous return, the treatment measures of increasing cerebral perfusion pressure is seen to be an inducer of further aggravation of cerebral edema, thus giving rise to the risk of rebleeding. It follows that if the cerebral vein also has a contractile spasm, it can only be treated by vasodilation.

### Selection of detection methods for cerebral vasospasm

Digital subtraction angiography (DSA) is treated as the “gold standard” for the diagnosis of cerebral vasospasm. The basic principle of DSA is described as follows: the angiographic X-ray fluorescence images of the inspected unit before and after the injection of the contrast agent are enhanced by an image intensifier and then scanned with a high-resolution television camera tube. The images are divided into small squares and made into matrixes, forming a video image composed of the pixels in the small grids. After logarithmic amplification and modulus/number conversion, the digital images are formed and stored separately. Then, the digital information of the two images is subtracted to obtain the difference signals of different values. Finally, *via* contrast enhancement and number/modulus conversion, the subtraction of vascular images are thus obtained and displayed on the screen, from which the bone, muscle, and other soft tissues are removed. But, with respect to small animals, this method is difficult to operate and control the dose of angiography, giving rise to high mortality, which is therefore not suggested to be repeated in large quantities.

Transcranial Doppler ultrasound (TCD) uses pulse Doppler technology and 2 MHz emission frequency to make the ultrasonic beam penetrate the thin part of the skull and directly record the Doppler signal of cerebral artery blood flow, so as to obtain the hemodynamic parameters of the cerebral basilar artery and reflect the state of cerebrovascular function. To be specific, the imaging principle is to diagnose the degree of cerebral vasospasm according to the blood flow velocity of the measured blood vessels, but the blood flow velocity is affected by many factors. Therefore, the estimation of cerebral vasospasm may be insufficient if the measured blood flow velocity is used to diagnose cerebral vasospasm only (27). For New Zealand rabbits, it is also difficult to find a suitable weak part of the skull to pass through the ultrasound.

Magnetic resonance imaging (MRI) is a kind of imaging technology that uses the hydrogen nuclei (proton) in human tissue to be excited by a radio frequency pulse in a magnetic field to produce a magnetic resonance signal, and reconstruct the image of a certain level of the human body through computer processing. It therefore possesses merits, such as safety, non-invasion, and contrast agent free. Besides, SWI sequence is a new MRI technology developed in recent years, which owns high sensitivity to deoxyhemoglobin in venous vessels. It has the advantages of high resolution and high signal-to-noise ratio (28). In addition, the TOF-MRA sequence is very sensitive to blood flow velocity and vessel routing and is also ideal for displaying small arteries.

### Spasmodic changes in deep cerebral veins

The basilar artery has always been the critical target vessel in the study of cerebral vasospasm after SAH (6–10). Basilar artery diameter in the SAH group was (0.66 ± 0.06) mm on 0 d and (0.44 ± 0.07) mm on 5th d after modeling. The statistical difference indicated that the model was successful. Meanwhile, the average diameter of the internal cerebral vein and basilar vein in this group before modeling was (0.57 ± 0.13) mm and (0.38 ± 0.08) mm, respectively. At the same time, it was only (0.42 ± 0.08) mm and (0.25 ± 0.12) mm on the 5th d after modeling. Compared with that before modeling, the diameter of the internal cerebral vein and basilar vein in the SAH group decreased by 26 and 34%, respectively. Accordingly, it can be confirmed that the deep cerebral veins and basilar artery did have spasmodic contractile changes after SAH. However, there was only 1 case each (6.7%) of mild, moderate, and severe stenosis of the great cerebral vein. This result may be due to the fact that there is no clear boundary between the great cerebral vein and the straight sinus in MRI and microanatomic observation. In the MRI, the exact location of the great cerebral vein is difficult to determine. The diameter measured in this study is more likely to be the diameter of the straight sinus. The sinus wall is made of dense collagen fiber composition, which is inflexible and inelastic, and is not prone to spasmodic changes.

To find out the time change regulation of cerebral deep vein spasm, this study selected the internal cerebral vein and basilar vein as the observation objects for continuous magnetic resonance detection, and compared them with basilar arterial spasm. According to the results, the basilar artery of the model well simulated the pathological process of cerebral vasospasm after SAH: obvious spasmodic changes occurred during 3rd−5th d after modeling, and the stenosis continued till the end of the 7th d, gradually approaching to that before modeling. This change in regulation was consistent with the report of Tsurutani et al. (29). However, the internal cerebral vein showed significant stenosis on 1st d after modeling, recovered on 3rd d, showed evident stenosis again on 5th d, and gradually approached normal until the end of 9th d. The results suggest that the diameter of the internal cerebral vein also changes regularly after SAH in rabbits, and its occurrence time and regularity are inconsistent with those of the basilar artery.

### Is spasmodic changes in deep cerebral veins vasospasm?

#### Analysis of stenosis degree

There may be two kinds of spasmodic changes in the diameter of deep cerebral veins in rabbits after SAH by SWI sequence: one is vasospasm caused by the contraction of the smooth muscle of the venous wall under the action of various physical and chemical stimuli; the other being that the diameter of the venous vessel becomes smaller, the volume of blood supply decreases after the vasospasm, and the venous blood flow playing the role of passive drainage also reduces, thus showing “pseudospasm” on MRI.

This study first compared the average diameter of blood vessels before modeling (0 d) with that of 1, 3, 5, 7, 9, and 11 d after modeling in the SAH group, and then subtracted the average diameter of each time point after modeling from the data before modeling. Then, the result was divided by the average diameter before modeling, and the degree of stenosis of each vessel was obtained. Hence, by comparing the stenosis degree of arterial and venous vessels, we analyzed whether the spastic changes of deep cerebral veins were an inducer of passive stenosis caused by the decrease of arterial blood supply.

By comparing the stenosis degree of the internal cerebral vein, basilar vein, and basilar artery at each time point, it is evident that there is no correlation between the stenosis degree of the artery and vein. Besides, the stenosis degree of the internal cerebral vein and basilar vein at each time point was seemingly similar on 1st d (18.86 and 18.42%, respectively). In comparison, the spasm degree of the basilar artery was only 3.03%. Furthermore, the stenosis degree of the internal cerebral vein and the basilar vein on the 3rd d was 13.21 and 23.68%, respectively. Both the values were much <36.36% observed for the basilar artery. What is more, on 5th d, the stenosis degree of the internal cerebral vein and basilar vein reached a peak of 33.96 and 34.21%, respectively. At this time, the basilar artery still retained the spasmodic peak of the 3rd d (33.33%), whose change was not noticeable. Although the stenosis degree of the internal cerebral vein and basilar vein recovered on the 7th d, it was still serious (28.30 and 28.95%, respectively). At this time, the spasm degree of the basilar artery was significantly reduced to 13.64%. On 9th d, the stenosis degree of the internal cerebral vein and basilar vein reduced by 15.09 and 21.05%, respectively, while the spasm degree of the basilar artery was only 9.09%. Finally, all blood vessels had recovered or nearly returned to normal on the 11th d.

According to the results mentioned above, except for 11th d, the stenosis degree between the cerebral vein and artery was significantly different on 1st, 3rd, 5th, 7th, and 9th d after modeling, which was obviously inconsistent with the conjecture of “ pseudospasm”.

#### Analysis of change rate

After analyzing the spasticity degree of the internal cerebral vein, basilar vein, and basilar artery, this study analyzed the measured value from another angle to more intuitively show the fluctuation in arteriovenous diameter between two adjacent time points and to further confirm that the spasmodic change is spasticity. To be specific, the change rate of vascular diameter was obtained by subtracting the previous measurement value from the previous one in the SAH group, and then dividing the previous value. If the results are positive, it indicates a reduction, while a negative value exhibits an enlargement.

The change in the basilar vein was 18.89% smaller than that observed before modeling, and it was similar to that of the internal cerebral vein, and the change in the basilar vein was 18.42% compared with that of 0 d, while the change in the basilar artery 1 d after modeling was not obvious (only 3.03%). On 3rd d, the diameters of the internal cerebral vein, basilar vein, and basilar artery were significantly different; the internal cerebral vein expanded by 6.98%, and the basilar vein continued to shrink by 6.45%. However, the basilar artery was significantly reduced, with a change rate as high as 34.38%; the change rate of internal cerebral and basilar veins was the most obvious on 5th d. On 7th d, the change rates of the internal cerebral vein and basilar vein were similar, which were −8.57% and −8.00%, respectively, while the diameter of the basilar artery expanded more, reaching −29.55%. On 9th d the change rate of the internal cerebral vein was −18.42%, the change rate of the basilar vein was −11.11%, and the change rate of the basilar artery was −5.26%. On 11th d the diameter of the internal cerebral vein and basilar vein was further enlarged, and the change rate was −13.33 and −20.00%, respectively, and that of the basilar artery was 10.00%.

The change rate reflects the fluctuation in vessel diameter in each time period. If the decrease in blood supply is caused by arterial spasm, the fluctuation in the vessel diameter of the internal cerebral vein and basilar vein should be consistent with that of the basilar artery. In addition, from the histological point of view, blood filling in the blood vessels leads to an increase in the gap between endothelial cells and the stretch of elastic fibers. The blood vessels can be expanded to a certain extent, and then the diameter of the vessels can be increased in the imaging observation. However, the decrease of blood flow in the blood vessels will only cause the relative collapse of the vessels, and there will be no obvious changes in the imaging. Moreover, the endothelial cells and elastic fibers will not be changed and there will be no contractile function, so spasm can only be the active contraction of smooth muscle. All the above perspectives reflect that the spasmodic changes of deep cerebral veins in this experiment are not caused by arterial spasms, but spastic changes are caused by spasticity.

In this study, artificial measurement is used to track the change in cerebral vessel diameter, which is time-consuming and laborious. In future research, artificial intelligence algorithms (30–32) will be tried in the recognition and measurement of deep cerebral veins. Computational simulations of the blood vessels (33, 34) can enable us to understand the blood dynamics and vascular structures better, which helps in the research of cardiovascular diseases.

## Conclusion

After inducing SAH in rabbits, the spasm was seen to occur in the basilar artery, and meanwhile, spastic changes were also observed in the deep cerebral vein. What is more, evident differences existed in the time regularity between the spasm and spastic change, thereby indicating that the spastic change of the deep cerebral vein turned into active contraction. In the clinical treatment of SAH, attention should be paid not only to the arterial spasm, but to the spasm of deep cerebral veins due to the fact that if the degree of cerebral deep vein spasm reaches more than 30%, it will seriously affect the cerebral venous return. Accordingly, the corresponding treatment measures should be taken according to the different time points of vasospasm. Meanwhile, the relationship between spasmolysis and cerebral perfusion pressure should be balanced when the spasm occurs simultaneously in the cerebral artery and deep cerebral vein.

## Data availability statement

The raw data supporting the conclusions of this article will be made available by the authors, without undue reservation.

## Ethics statement

The animal study was reviewed and approved by Ethics Committee of Anhui Medical University (Hefei, China, 2012238).

## Author contributions

XD contributed to conception and design of the study. ZZ, WZ, and YouyZ conduct animal experiments. YZ and YouzZ conduct the MRI. QF performed the statistical analysis. ZZ wrote the first draft of the manuscript. All authors contributed to manuscript revision, read, and approved the submitted version.

## Funding

This work was supported by the National Natural Science Foundation of China (81200895), the Natural Science Research Project of Anhui Colleges and Universities (KJ2021A1369), and the Support Program for Outstanding Young Top Talent in Anhui Colleges and Universities (gxyq2019208).

## Conflict of interest

The authors declare that the research was conducted in the absence of any commercial or financial relationships that could be construed as a potential conflict of interest.

## Publisher's note

All claims expressed in this article are solely those of the authors and do not necessarily represent those of their affiliated organizations, or those of the publisher, the editors and the reviewers. Any product that may be evaluated in this article, or claim that may be made by its manufacturer, is not guaranteed or endorsed by the publisher.
